# Sugar-added beverages consumption among kindergarten children of Crete: effects on nutritional status and risk of obesity

**DOI:** 10.1186/1471-2458-8-279

**Published:** 2008-08-06

**Authors:** Manolis Linardakis, Katerina Sarri, Maria-Styliani Pateraki, Manolis Sbokos, Anthony Kafatos

**Affiliations:** 1 Preventive Medicine and Nutrition Clinic, Faculty of Medicine, University of Crete, Greece

## Abstract

**Objective:**

To assess the intake of sugar-added beverages such as soft drinks and commercially available fruit juices in kindergarten children, and to examine its association with obesity indices, physical activity levels and dietary habits.

**Methods:**

A total of 856 children aged 4–7 years living in Crete, Greece in 2004–5 were included in this cross-sectional study. Nutrient and food intake was assessed with the use of 3-day weighed food records. Body measurements were used in order to assess BMI and waist circumference, and moderate-to-vigorous physical activity was calculated with the use of a questionnaire.

**Results:**

Approximately 59.8% of all children consumed sugar-added beverages on a daily basis. High intake of sugar-added beverages (> 250 g/day) was associated with low intakes of calcium (p < 0.001), vitamin A and E (p < 0.010), fruits and vegetables (p = 0.007), and milk and yogurt (p = 0.048). Compared to non or low consumers, high consumers of sugar-added beverages (> 250 g/day) had higher BMI levels and two times greater risk of being overweight and/or obese (OR:2.35, p = 0.023).

**Conclusion:**

High intake of sugar-added beverages in kindergarten children is associated with poor eating habits and inadequate nutrient intake, as well as increased risk for developing childhood obesity.

## Background

Increasing rates of childhood obesity, starting from preschool age, is a worldwide phenomenon [1-3]. In Greece it is estimated that the overall prevalence of overweight and obesity in school children aged 6–17 years old is 17.3% and 3.6% respectively [4], whereas for preschool children (1–5 years old) it is 14.2% and 7.5% respectively [5].

Increased intake of sugar-sweetened beverages, such as soft drinks and commercially available fruit juices, amongst a number of other factors such as fast food eating, breakfast skipping, inactivity, and increased TV viewing hours, is thought to contribute to weight gain and risk of obesity in childhood and adolescence [6-12]. These data are consistent with the findings in the adult population where increased intake of sweetened beverages is associated with weight gain and increased risk of chronic diseases such as diabetes [13,14].

A number of studies have suggested a potential association between soft drink intake and prevalence of childhood obesity [15]. The data for Greece is, however, limited. The 2000–2001 World Health Organization Report stated that less than 20% of Greek adolescents were daily soft-drink consumers, placing Greece very low in the worldwide ranking of soft-drink consumption. However, the Greek Health-Behavior-in-Schoolaged-Children study indicated that more than half of 11–15 year olds consume soda drinks on a daily basis [16]. Only a few studies are available on Greek children's dietary habits including soft drink consumption, but none of these covered kindergarten children. General trends are indicated by the National Statistical Service of Greece, which reported a striking increase of soft-drink consumption per person per year from 17.2 L in 1972 to 44.6 L in 2005 , while overall consumption of soft drinks and other sweetened beverages was 60 L per capita in 2005.

The aim of the present study was to assess the intake of sugar-added beverages such as soft drinks and commercially available fruit juices in kindergarten children of Crete, and to examine its association with obesity indices, physical activity levels and dietary habits.

## Methods

### Study population

The study was conducted in 2004–5 in all public kindergartens of the county of Chania in Crete. The age range of kindergarten children recruited in this study was 4–7 years of age. The present study is part of a larger intervention study [17]. It involves the baseline measurements performed in kindergarten children in order to assess their nutritional and health status before entering a nutrition education intervention study.

Out of 2630 kindergarten children, 1988 accepted to participate (77%). Another 233 children did not participate in the measurements either because they were not present at school or they were sick on the day of the examination. Finally, the 3-day weighed food records were returned for 856 (49%) children and were included in the analyses of the present study.

Parents gave written consent for the children's participation after having been fully informed both orally and in writing. The study was approved by the Greek Ministry of Education and the Ethics Committee of the University of Crete.

### Dietary intake assessment

Three-day weighed food records were completed by the children's parents after they were given detailed oral and written instructions. Food records included two work days and one weekend day. Brands of products as well as methods of preparation were also recorded. Nutrient and food groups' intake was assessed as the mean of the 3-day weighed food records. The food group of sugar-added beverages included soft drinks and commercially available fruit juices with added sugar (excluding isotonic drinks).

The dietary software "Greek Diet", used to calculate dietary intakes, was initially created in 1990 by the Preventive Medicine and Nutrition Clinic of the University of Crete and later, in 1998, was upgraded using the USDA Nutrient Database for Standard Reference (release 11, 1996; USDA Agricultural Research Service, Washington, DC, USA). An extensive description of the database is given elsewhere [18].

### Physical activity questionnaire

A physical activity questionnaire, completed by the parents, was used to assess moderate-to-vigorous physical activity (MVPA). MVPA was defined as continuous vigorous activity causing sweating and heavy breathing for periods longer than 15 minutes, but with occasional breaks in intensity, rather than the strict aerobic definition of 20 continuous minutes appropriate to older children and adults [19]. Parents were questioned about the frequency, intensity and duration of any regular physical activity of their child (average minutes per week) as well as registration in any after-school physical activity-related classes. Information on sedentary behavior such as TV viewing or computer use was also recorded. The questionnaire used has been validated by heart rate monitoring [20].

### Body measurements

Length and height of children were measured to the nearest 0.1 cm using a short stadiometer for children > 6 years of age, and in the supine position for children < 6 years of age. Weight in light clothes was measured to the nearest 0.1 kg on a portable electronic scale (Seca model 770). Waist circumference (WC) was measured at the level of umbilicus at minimal respiration to the nearest 0.1 cm.

#### Defining adiposity status

BMI was calculated as body weight (kg) divided by height (m) squared. Subjects were classified as overweight and obese, according to the cut-offs for childhood overweight and obesity adopted by the International Obesity Task Force [21]. With regard to WC, children were categorized according to the 90^th ^percentiles of Cretan children for their gender and age [22]. Children above the 90^th ^percentile of WC were classified as overweight and obese.

### Statistical analysis

The SPSS, version 15.0, statistical program was used for all analyses.

To assess for potential selection bias, we compared the basic characteristics between the children who returned the 3-day weighed food records and those who did not (856 vs 879). There was no significant difference in the distribution of gender (χ^2 ^test, p = 0.227), age (student t-test, p = 0.387) and BMI (student t-test, p = 0.147) between responders and non-responders.

Chi-square (χ^2^) test was used for detecting differences in main characteristics between two genders. Intake of sugar-added beverages was divided into four categories: a. no consumption, b. < 150 grams/day, c. 151–250 grams/day and d. > 250 grams/day. Analysis of Covariance (linear trend) was used to estimate increase in nutrient intake, food consumption, body measurements and MVPA levels within the four groups (heterogeneity was tested by Levene's test).

Logistic regression analysis was used for estimating the risk (Odds Ratios-OR) of being overweight and/or obese (International Obesity Task Force criteria for BMI and > 90^th ^sex-age percentiles for WC) in relation to sugar-added beverage consumption. The reference category was children that were non-consumers. Gender, age, BMI, energy intake and birth weight were used as covariates.

## Results

Table 1 shows that overall 19% (n = 160) and 10.8% (n = 91) of the children were overweight and obese, respectively. Boys had significantly higher energy intake than girls (p < 0.05), whereas no other differences were found with regard to BMI, MVPA and intake of sugar-added beverages between the genders. Overall, 59.8% of all children were regular consumers of sugar-added beverages.

**Table 1:** Demographic characteristics of the study population

	**Boys**	**Girls**
	447 (52)^1^	409 (48)
**Kindergarten**^1^		
1^st ^class	184 (52)	171 (48)
2^nd ^class	263 (52)	238 (48)
		
**Age **(years)	5.4 ± 0.6 (447)^2^	5.4 ± 0.6 (409)
		
**Area of residence**^1^		
Urban	179 (40)	168 (41)
Sub-urban	21 (5)	33 (8)
Rural	246 (55)	208 (51)
		
**Body Mass Index **(kg/m^2^)	16.7 ± 2.2 (441)^2^	16.6 ± 2.9 (402)
Overweight^1^	78 (17.6)	82 (20.4)
Obese^1^	46 (10.4)	45 (11.2)
		
**Energy **(kcal)^3^	1828 ± 388 (447)^2^	1686 ± 377 (409)
		
**MVPA **(mins/week for exercisers)^4,5^	184 ± 85 114 (25.5)	177 ± 104 156 (38.1)
		
**Sugar-added beverages **(g/day)^5^	181 ± 131^2^	162 ± 136
Consumers	260 (58.2)^1^	252 (61.6)

1 Chi-square test (χ^2^) [Values are presented as N(%)].

2 Analysis of variance [Values are presented as Mean ± SD (N)].

3 p-value < 0.05

4 Moderate Vigorous Physical Activity

5 Children with zero minutes of activity (MVPA) and zero consumption of sugar-added beverages were not included.

Table 2 shows that those in the highest category of sugar-added beverage intake also had higher intake of total energy, carbohydrate and vitamin C (p for trend < 0.001) than non- or low-consumers. Furthermore, high consumers of sugar-added beverages consumed significantly less calcium, vitamin A and E (p for trend < 0.010), fruit and vegetables (p for trend = 0.007), milk and yogurt (p for trend = 0.048), and olive oil (p for trend = 0.008). Intake of sugar-added beverages was positively associated with intake of sugar not contained in beverages and sweet and/or savory snacks (p for trend = 0.003 and 0.041, respectively). Table 2 also shows that high consumers of sugar-added beverages had higher levels of BMI (p for trend = 0.003), whereas MVPA did not differ between all types of consumers and non-consumers.

**Table 2:** Diet, body measurements and physical activity levels among non-, low- and high consumers of sugar-added beverages of kindergarten children of Crete.

		**Consumption of Sugar-added beverages**					
				
		**Non consumers**	**Consumers**				
					
			< 125 g/d	125–250 g/d	> 250 g/d		
				
		Mean ± Standard Error (N)				***P*-value**	***P*-value **for trend
**Nutrients**							
**Energy **(kcal)		1682 ± 20 (339)	1749 ± 24 (244)	1850 ± 29 (164)	1915 ± 38 (97)	**<0.001**	**<0.001**
**per 1000 kcal**							
	**Proteins **(g)	37.1 ± 0.3 (339)	35.3 ± 0.4 (244)	34.6 ± 0.4 (164)	32.9 ± 0.6 (97)	**<0.001**	**<0.001**
	**Carbohydrates **(g)	107 ± 1 (339)	111 ± 1 (244)	115 ± 1 (164)	122 ± 1 (97)	**<0.001**	**<0.001**
	**Saturated fat **(g)	17.8 ± 0.2 (339)	17.5 ± 0.2 (244)	17.0 ± 0.2 (164)	15.9 ± 0.3 (97)	**<0.001**	**<0.001**
	**Mono-unsaturated fat **(g)	19.5 ± 0.2 (339)	18.7 ± 0.2 (244)	18.6 ± 0.3 (164)	17.7 ± 0.4 (97)	**<0.001**	**<0.001**
	**Poly-unsaturated fat **(g)	5.3 ± 0.1 (339)	5.2 ± 0.1 (244)	4.9 ± 0.1 (164)	5.1 ± 0.2 (97)	0.077	0.109
	**Calcium **(mg)	614 ± 9 (339)	581 ± 11 (244)	562 ± 13 (164)	491 ± 17 (97)	**<0.001**	**<0.001**
	**Vitamin A **(μg)	400 ± 14 (339)	413 ± 17 (244)	349 ± 21 (164)	338 ± 27 (97)	**0.021**	**0.010**
	**Vitamin E **(mg)	3.5 ± 0.1 (339)	3.5 ± 0.1 (244)	3.3 ± 0.1 (164)	3.1 ± 0.1 (97)	0.078	**0.010**
	**Vitamin C **(mg)	54.2 ± 1.7 (339)	55.3 ± 2.0 (244)	68.6 ± 2.4 (164)	82.1 ± 3.2 (97)	**<0.001**	**<0.001**
**Food groups **(g/day)							
**Cereals, rice, potatoes**		167 ± 5 (339)	174 ± 6 (244)	186 ± 7 (164)	177 ± 9 (97)	0.156	0.198
**Fruits and Vegetables**		214 ± 7 (319)	186 ± 8 (239)	189 ± 10 (161)	171 ± 13 (94)	**0.008**	**0.007**
**Milk and yogurt**		336 ± 9 (336)	339 ± 12 (242)	327 ± 14 (162)	297 ± 18 (96)	0.238	**0.048**
**Cheese**		28 ± 1 (291)	29 ± 1 (218)	29 ± 1 (153)	28 ± 1 (83)	0.978	0.699
**Red meat**		52 ± 2 (293)	55 ± 3 (215)	52 ± 3 (153)	60 ± 4 (93)	0.261	0.103
**Olive oil**		17 ± 1 (302)	14 ± 1 (222)	14 ± 1 (152)	13 ± 1 (86)	0.154	**0.008**
**Sugar and sweets **(excluding sugar from sugar-added beverages)		22 ± 1 (248)	23 ± 2 (208)	26 ± 2 (129)	27 ± 3 (77)	0.066	**0.011**
**Snacks**		27 ± 2 (165)	27 ± 2 (149)	29 ± 3 (90)	34 ± 3 (67)	0.232	**0.041**
**Body measurements**							
**Weight **(kg)		22.4 ± 0.2 (308)	22.1 ± 0.3 (228)	22.7 ± 0.3 (156)	23.2 ± 0.4 (91)	0.258	0.057
**Body Mass Index **(kg/m^2^)		16.6 ± 0.1(308)	16.4 ± 0.2 (228)	16.9 ± 0.2 (156)	17.4 ± 0.3 (91)	**0.028**	**0.003**
**Waist circumference **(cm)		55.7 ± 0.3(308)	55.6 ± 0.4 (228)	56.4 ± 0.5 (156)	56.1 ± 0.6 (91)	0.570	0.335
**Physical activity**							
**MVPA **(mins/week)		63 ± 6 (326)	53 ± 6 (242)	68 ± 8 (163)	44 ± 10 (96)	0.137	0.246

Analysis of Covariance (Heterogeneity was tested by Levene's test). Gender, age and BMI were used as covariates in the analysis of nutrients and foods; gender, age, energy intake and birth weight were covariates in the analysis of body measurements; gender and age were covariates in the analysis of MVPA.

**Figure 1:**
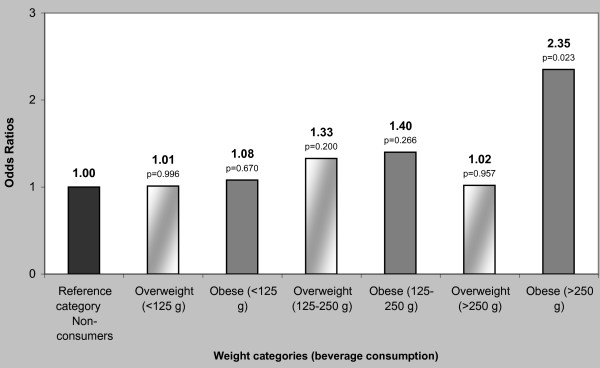
**Risk (odds ratio) for Body Mass Index of kindergarten children of Crete in relation to consumption of sugar-added beverages**. Logistic regression analysis. Gender, age, energy intake and birth weight were used as covariates. BMI cut-offs for overweight and obesity were determined by International Obesity Task Force criteria.

**Figure 2:**
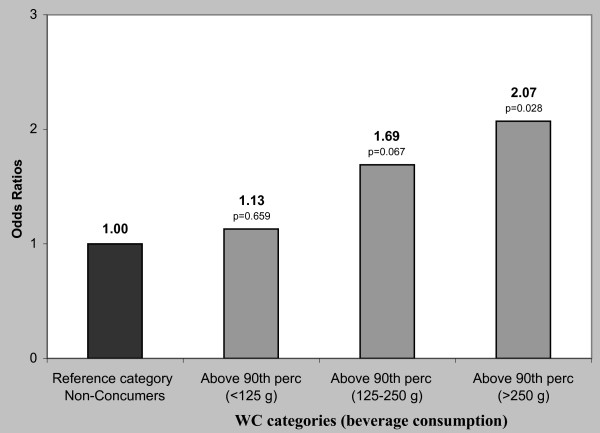
**Risk (odds ratio) for Waist circumference of kindergarten children of Crete in relation to consumption of sugar-added beverages**. Logistic regression analysis. Gender, age, energy intake and birth weight were used as covariates. The 90th percentiles of waist circumference were estimated by gender-age categories.

Children consuming > 250 g of sugar-added beverages per day had two times higher risk of being obese according to their BMI (OR = 2.35, p = 0.023) and WC (OR = 2.07, p = 0.028), when compared to non-consumers with BMI and WC within the normal ranges (Figures 1 and 2).

## Discussion

In the present study almost 30% of kindergarten children were overweight and/or obese, whereas 60% of the children were daily consumers of sugar-added beverages.

In agreement with other studies [14,23,24], the present study indicates that intakes of milk and yogurt, calcium and vitamin A decrease as sugar-added beverages become a favorite choice of children on a daily basis (> 250 g/d). This finding points to a significant risk factor for impaired calcification of growing bones, since high phosphate levels of soda drinks could affect calcium metabolism. The importance of adequate calcium intake from an early age is underlined by the fact that it can prevent bone loss and osteoporosis in adult life [25-30].

Low fruit and vegetable consumption in high consumers of sugar-added beverages led to low intake of vitamin A and E. Such an effect could have adverse health effects in the long-term, since fruit and vegetable consumption is inversely associated with some types of cancer, diabetes and heart diseases [31-33]. More importantly 'healthy' dietary habits established in early childhood contribute to similar habits later in life and influence adult health [34,35].

High vitamin C intake of high sugar-added beverages consumers could be explained by the fact that commercially available sweetened fruit juices are usually enriched with vitamins.

Risk of overweight and obesity in high consumers of sugar-added beverages was twice as high as in low- or non-consumers. Increased total energy intake and similar levels of MVPA could explain the increased risk of obesity in high-consumers as compared to non- or low-consumers. Analysis of the National Health and Nutrition Examination Survey data [36] regarding preschool children showed higher daily energy intake for those consuming sweetened fruit juices and sugar-added soft drinks, and a positive association between soft drink intake and overweight [12]. In this context, other studies have shown that soft drink consumption is related with short stature and obesity in preschool children [8], as well as weight gain [7] and increased risk of obesity in school children [37]. In particular, risk of obesity was found to increase by 60 % for each serving of sweetened beverages in adolescents [37], whereas overweight preschool children consuming soft drinks were more likely to retain their increased weight [38]. Moreover, James et al. showed that after 1 year of a school based education program on nutrition children reduced their intake of soft drinks and prevalence of overweight declined [39].

## Conclusion

In conclusion, the present study indicates that high intake of sugar added beverages is associated with unfavourable nutritional status as well as high levels of obesity in kindergarten children of Crete. It seems that in Greece there is limited awareness of the potential risks to children's health that could be related to the unrestricted intake of sugar-added beverages. Policies on banning sugar-added beverages being purchased at schools as well as implementing nutrition education programmes, which have been adopted elsewhere in Europe, could be beneficial in the battle against rising prevalence of childhood overweight and obesity in Crete.

## Competing interests

The authors declare that they have no competing interests.

## Authors' contributions

ML performed all statistical analysis and wrote the section of statistics. KS wrote the manuscript. MSP and MS performed the computing of data and did part of the bibliographic research, AK conceived of the study, is the head supervisor and reviewed the paper. All authors read and approved the final manuscript.

## Pre-publication history

The pre-publication history for this paper can be accessed here:


